# The Effect of a Combination of Vibration and External Cold on Pain Caused during Vaccine Injection in Infants: A Randomized Clinical Trial

**DOI:** 10.1155/2024/7170927

**Published:** 2024-03-04

**Authors:** Zahra Unesi, Zahra Amouzeshi, Javad Jamavar, Fatemeh Mahmoudzadeh Zarandi

**Affiliations:** ^1^Faculty of Nursing and Midwifery, Birjand University of Medical Sciences, Birjand, Iran; ^2^Department of Nursing, School of Nursing and Midwifery, Cardiovascular Diseases Research Center, Birjand University of Medical Sciences, Birjand, Iran; ^3^Science and Research Branch Islamic Azad University of Medical Sciences, Birjand, Iran; ^4^Nursing Research Center, Kerman University of Medical Sciences, Kerman, Iran

## Abstract

**Aim:**

This study was conducted to determine the effect of combining vibration and external cold on pain caused by vaccine injection among six-month-old infants.

**Design:**

Randomized controlled trial.

**Methods:**

In this clinical trial, 80 eligible infants were selected from the infants referred to a health center as per the inclusion criteria. The infants were assigned to either a control group or an intervention group by block randomization. In the intervention group, a vibrating and cold device was placed above the injection site from one minute before to 15 seconds after the pentavalent vaccine injection. In the control group, no intervention was performed, and they were vaccinated according to the routine procedure. The pain status in the two groups was measured using the Modified Behavioral Pain Scale (MBPS) 15 seconds after the injection, and the crying duration was assessed from the injection of the vaccine till the end of it. Data were analyzed in SPSS 23 software using Mann–Whitney, *t*, Spearman, and chi-square tests. The level of significance was set to *p* < 0.05.

**Results:**

Most participants in the control (55%) and intervention (55%) groups were girls. Statistical data analysis of 80 infants showed that the mean pain intensity (*p* = 0.032) and duration of crying (*p* = 0.0001) in the intervention group (6.1 ± 1.8, 32.47 ± 16.78) were lower than those of the control group (7.2 ± 0.1, 51.02 ± 25.9), respectively.

**Conclusion:**

Because the intensity of pain, especially the duration of crying, was lower in the intervention group than in the control group, we may suggest that nurses use simple pain relief solutions in vaccination centers, such as a combination of vibration and cold. This trial is registered with IRCT201207157130N2.

## 1. Introduction

Pain is defined by the International Association for the Study of Pain as “An unpleasant sensory and emotional experience associated with actual or potential tissue damage” [1]. Immunizations and other medical procedures involving needles are the most common and significant causes of pain in children [2]. Due to the absence of inhibitory systems and the short pain pathway, infants are more sensitive to pain and react more severely than older children [3].

Uncontrolled pain in infants causes physiological, hormonal, and behavioral responses that may cause irreparable damage in the short and long term, including apnea, cardiac arrhythmia, increased intracerebral pressure, increased blood pressure, tachypnea, immunosuppression, endocrine dysfunction, and a delay in the development of the nervous system and wound healing [4].

Pain relief is conducive to the degree of pain that infants may perceive or experience through subsequent injection sessions [5]. Injection pain relief during infancy alleviates the negative emotional side effects that injection may bring about for the parents and the child [6]. It can also enhance parental compliance with the immunization program [7].

Nurses must be able to manage painful procedures to lessen emotional and physical effects on children [2]. Alongside this, pharmaceutical and nonpharmacological interventions should be an integral part of nursing practice [8].

Nonpharmacological and pharmacological methods, such as local anesthetic creams, vapor coolant (cold) spray, pinching, rubbing, vibration near the injection site, parent education, and distraction, are employed to manage pain caused by childhood vaccinations [9, 10]. Nonpharmacological methods are the most effective way to control and tolerate pain caused by minimally invasive procedures, as they can replace a sense of control for the helplessness associated with pain [11].

The gate control theory of pain is built on to explain the efficacy of the majority of these nonpharmacological methods; for example, skin stimulation and mental distractions can stimulate the activity of the thalamus and cerebral cortex, resulting in the closure of the gate and interruption of the transmission of the pain message to the brain. This, in turn, lowers pain perception. Golianu et al. assert that stimulating infants' different senses through massage, sucking, taste, cold, and touch, alone or in combination, blocks pain gates based on the gate control theory [12]. Research has shown that the combination of cold and vibration can effectively reduce pain in children during invasive procedures [2, 10, 13, 14].

Despite the increased utilization and development of pharmacologic and nonpharmacologic interventions, pain management remains suboptimal. This suggests that the evidence is not being implemented in clinical settings or that healthcare providers are underutilizing the interventions. Therefore, it is essential to equip healthcare professionals, especially nurses, with interventions that are likely to be routinely implemented in clinical settings [8].

Considering the affordability and nonintrusive nature of cold and vibrating skin stimulation, as well as the limited research on the combination of vibration and cold in infants, a study was conducted titled “The effect of a combination of vibration and external cold on pain caused during vaccine injection in infants.”

## 2. Materials and Methods

### 2.1. Trial Design

This study was designed as a randomized (1 : 1) open-label study and was performed in two groups in parallel. The study was conducted in a health center located in Birjand, South Khorasan, Iran. In this randomized clinical trial, the CONSORT reporting guideline is followed. For detailed information, see the supplementary material.

### 2.2. Participants

The inclusion criteria comprised parental consent for participation, the infant being awake and calm, the infant's diaper being dry, no history of hospitalization for illness or surgery, absence of cold or diarrhea at the time of vaccination, term at birth, no analgesic medication consumed by the mother and infant 48 hours prior to vaccination, the normality of the child's growth curve, the absence of cerebral palsy or mental retardation, and the habit of sucking a finger. The exclusion criterion consisted of the mother's refusal to continue participation.

### 2.3. Data Collection Tools

The data collection tools included a characteristics form, the Modified Behavioral Pain Scale (MBPS), and a stopwatch. The characteristics form included gender, infant weight, crying duration, the interval between injection and the latest sleep and feeding session before injection, and previous painful procedure experiences.

The MBPS was employed to assess infants' behavioral responses to pain. This three-domain scale measures an infant's facial expressions, body movements, and crying. Facial expressions include smiling (scored 0), neutral expression (scored 1), frowning, scared, or grimacing (scored 2), and furrowing eyebrows, closing eyes tightly, and opening the lips with or without reddening of the face (scored 3). Body movements include natural activity and movements (scored 0), resting and relaxed (scored 0), partial movements such as squirming, arching, limb tensing, and clenching, as well as attempting to avoid pain by withdrawing the limb where the injection is done (scored 2), agitation with complex or generalized movements involving the head, torso, or other limbs (scored 3), and body rigidity (scored 3). Lastly, the way the infant cries consists of laughing or giggling (scored 0), not crying (scored 1), moaning quietly, vocalizing gently, or whimpering cry (scored 2), full lunged cry or sobbing (scored 3), and full lunged cry more than baseline cry (score 4).

In this instrument, the scores for facial expressions and body movements range from 0 to 3, and those for crying range from 0 to 4. The total score is calculated by adding the scores of the three domains above. As such, the minimum possible score for the infant's behavioral response to pain is 0, and the maximum possible score is 10, with a higher score indicating greater pain experienced by the infant. MBPS is a standard tool. Numerous studies have repeatedly demonstrated this instrument's good reliability and validity [6]. The scale has been utilized in Iran by Taavoni et al. [15] and Hadadi Moghaddam et al. [16]. Inter-rater reliability was applied to determine the instrument's reliability in the present study. Using the MBPS tool, two research assistants independently measured the behavioral responses caused by pain in 15 infants. The correlation coefficient between these observations was high (kappa = 0.85).

The duration of crying was measured using a Samsung mobile phone chronometer. Before sampling, the phone chronometer's accuracy was confirmed by comparing it to that of a trusted chronometer.

### 2.4. Intervention

After obtaining the required permits to conduct the intervention, the researcher visited the intended health center. Based on the inclusion criteria, the research sample was selected from the children referred to the center. They were subsequently assigned at random to either the control or intervention group using the alternating blocking method. The sampling period lasted five months. After the infants were assigned to the intervention group, they were placed on the vaccination bed in a quiet environment with their mother present. Skin stimulation was performed by a cold vibrating device with 100 Hz vibration at a distance of half a centimeter above the injection site for one minute prior to injection and 15 seconds after [17, 18]. The device is a compact, durable plastic device measuring 5 × 4 × 2.5 cm. The device consists of two main parts. First, there is the body, which vibrates at 100 Hz and can provide continuous or intermittent vibration when activated. Second, there is a detachable cooling pad that contains nontoxic gel. This pad can be frozen and will stay frozen for about 10 minutes at room temperature. It should be positioned half a centimeter above the injection site.

The same vaccinator injected pentavalent vaccine at a dose of 0.5 cc under the same conditions (ambient temperature, type of injection solution, temperature of injection solution, light, and sound) with the same implements in the depth of the vastus lateralis muscle. In the control group, vaccinations were administered according to the center's routine protocol [18].

### 2.5. Outcomes

At the same time that the vaccinator administered the vaccine, a research assistant who was unaware of the research hypotheses recorded pain intensity 15 seconds after the injection using the MBPS criteria. Another fixed research assistant measured the crying duration in seconds from when the needle was inserted to three minutes after the injection.

### 2.6. Sample Size

According to a similar study [18] using the formula (*z*(1 − *α*/2) + *z*(1 − *ß*))2 (*s*12 + *s*22)/(*m*1 − *m*2)2, the sample size per group was computed as *n* = 18, given *m*1 = 8, *m*2 = 9.6, *s*1 = 0.81, *s*2 = 1.2, a 95% confidence, and 80% test power (based on the mean and standard deviation of the pain intensity of the intervention and control groups). Nevertheless, in order to increase the accuracy and validity of the study, the number of samples in each group was decided to be *n* = 40.

### 2.7. Data Analysis

The data were analyzed using SPSS software (IBM Corp.), version 23. The demographic characteristics of the infants were described using descriptive statistics (mean, standard deviation, and frequency). The Kolmogorov–Smirnov test was used to study data distribution. A chi-square test was utilized to compare the gender of infants in the control and intervention groups. In addition, the independent *t*-test was employed to compare weight and crying duration. The Mann–Whitney test was utilized due to the nonnormality of the data distribution for the variables of pain intensity and the time interval between the injection and the latest feeding and sleep before the injection. Lastly, the relationship between pain intensity and crying duration was determined using Spearman's correlation coefficient. Also, the effect size was calculated. The significance level is considered less than 0.05.

### 2.8. Ethical Considerations

The research protocol was registered on the Iranian Registry of Clinical Trials website with the identifier IRCT201207157130N2. It was approved by the ethics committee affiliated with Birjand University of Medical Sciences with the identifier IR.BUMS.REC.1394.444. The study objectives were explained to parents, and informed consent was obtained. They were also assured that the data were confidential and they could withdraw from the study at any time.

## 3. Results

Eighty infants were enrolled in the study and equally divided into two groups: intervention and control. The data of 80 infants (40 in each group) were finally analyzed (flow diagram 1 (available here)). Most participants in the control (55%) and intervention (55%) groups were girls. According to the study's results, the variables of gender, feeding time before vaccination, previous pain experience, weight, and the latest sleep time before vaccination were not statistically different between the two groups (Table 1). In other words, before the intervention, the two groups were matched in terms of these variables.

The Mann–Whitney test revealed that the mean pain score and duration of crying were lower in the intervention group than in the control group (Table 2).

In the study of the relationship between pain intensity and crying duration, the Spearman test revealed a significant direct relationship between pain intensity and crying duration in both the intervention group (*r* = 0.52, *p*=0.001) and the control group (*r* = 0.47, *p*=0.002).

## 4. Discussion

The present study aimed to determine the effect of combining vibration and cold on infants' vaccination-induced pain. The results revealed that the intervention group experienced significantly less pain than the control group. In this regard, scientific theories postulate that various sensory stimuli compete with painful stimuli during painful procedures and prevent pain from being at the forefront of a person's consciousness, thereby altering the person's perception of pain [12]. Canbulat Sahiner et al. also demonstrated that the pain intensity in the group that received a combination of vibration and external cold was significantly lower than that in the control group receiving routine care. In the study by Canbulat Sahiner et al., the intervention group's mean pain intensity score on a 10-point scale was 3.54 points lower than that of the control group. In the present study, this score was only 0.9 points lower in the intervention group than in the control group. The difference in age groups may account for this difference. In Canbulat Sahiner et al.'s study, 7-year-olds were examined, whereas 6-month-old infants were analyzed in the current study. According to certain researchers, the sensitive skin of children under four years old may cause them to feel distressed when they touch something cold. Because of this, the effect of the intervention is likely to be less in younger children than in older children, but it does reduce the crying time [2].

The results of Canbulat et al. study revealed that a combination of skin stimulation with external cold and vibration could be used to alleviate children's pain and anxiety during immunization (in the age group of 7 years) [19]. Furthermore, in the study by Redfern et al., the effect of skin mechanical stimulation on the pain caused by vaccine injection in the 7-year-old age group was significantly less in the intervention group than in the control group, although it did not affect anxiety before the injection [20]. It was also discovered in studies by Canbulat Şahiner et al. that combining vibration and cold reduces the pain caused by intravenous injections [2, 10, 14]. In contrast, Benjamin et al., in a study involving 100 children aged 2 months to 7 years, concluded that vibration therapy does not alleviate the pain caused by the injection of the vaccine and that the intensity of the pain increases with age [5]. Notably, Benjamin's research focused solely on vibration. According to Golianu's theory, multiple sensory messages are more effective than single sensory stimuli in blocking pain gates [12]. In addition, Benjamin believed that the role of emotional and cognitive factors in modifying physical stimuli was responsible for the increased pain intensity in older age groups due to their increased awareness and cognition. According to him, the use of a vibrating device before injecting a child may remind the child of the impending injection, which may cause the child's pain to intensify [5]. In Benjamin's study, variables such as the vaccinator's variability, the type of vaccine, and the number of injections were not taken into account. Eden et al. believe that one of the factors influencing vaccine pain is its pH, such that the lower the pH of the vaccine is, the more pain a person experiences [7].

Most research on the effect of the specified intervention on intravenous injections has reported positive results, as evidenced by the review of the studies above. However, the number of research conducted in the field of vaccinations and intramuscular injections is limited, and in some instances, the results reveal inconsistent or even contradictory effects.

In the present study, the duration of crying was considerably shorter in the intervention group than in the control group. The crying duration index has been employed in addition to the pain measurement tool as a tool and a primary index to assess pain in numerous studies [4]. Moreover, research indicates that the duration of infants' cries in response to painful stimuli varies according to the intensity of the pain [4]. In support of this, the current study's findings revealed that pain intensity correlates directly with the duration of crying in both the intervention and control groups. In addition, the intervention group's mean pain score and crying duration were lower than those of the control group. In Unesi et al.'s study examining the effect of ShotBlocker on the pain caused by vaccine injection in 6-month-old infants, the mean pain intensity and duration of crying were lower in the intervention group than in the control group [21]. Nonetheless, some studies have produced contradictory results. For example, the studies conducted by Karaca Ciftci et al. evidenced that flick application and oral sucrose reduce pain-induced behavioral responses. However, they have only a moderate effect on reducing the duration of crying in the intervention group compared to the control group. Mowrey contends that the ineffectiveness of oral sucrose on the crying duration of infants results from the study participants not receiving a sufficient dose of oral sucrose [4, 6]. Furthermore, in the cited study, confounding variables such as sucking and mental distraction were not taken into account, which can interfere with the effect of the intervention on crying duration.

The study has several strengths. Firstly, the experimental and control groups were assigned in a randomized manner. Additionally, pain levels were assessed using pain scales that are both easily comprehensible and highly valid and reliable. Vibration and cold therapy is a cost-effective and reusable technology that requires minimal additional time, can be operated by parents instead of medical personnel, and potentially offers advantages for children of various ages. This study has confirmed that this method is suitable for regular use during immunization in infants.

One of the limitations of this study was the fact that it was conducted in a single center, which limits the ability to apply the findings to other contexts. It would be advisable to conduct further research with a larger sample size across multiple health centers. The current study's findings cannot be applied to infants and adolescents who are not in good health. Given the limited research in this area and the lack of knowledge among nurses about nonpharmacological pain interventions as the fifth vital sign, it is suggested that more research be done on age-appropriate interventions for children. Furthermore, it is crucial to conduct additional evidence-based studies across various painful interventions and age groups to establish the effectiveness of these methods. One of the limitations of this study was the potential bias in performance due to the open-label nature of the study. Meanwhile, an uninformed research assistant diligently documented pain intensity, while another dedicated research assistant meticulously tracked the duration of crying.

## 5. Conclusion

Considering the effect of vibration and external cold on the pain caused by vaccine injection in infants, it is recommended that health professionals utilize a combination of vibration and cold as a safe and simple method to lessen infants' pain and crying time during vaccination. This article contributes empirically to the existing literature on nonpharmacological methods of vaccination-induced pain relief in infants.

## Figures and Tables

**Table 1:** The demographic and medical characteristics of the 80 infants that participated in the study.

	Intervention group (*n* = 40)	Control group (*n* = 40)	*p* value	Effect size
Gender, *n* (%)			1.00^a^	0.00
Girl	22 (55)	22 (55)		
Boy	18 (45)	18 (45)		
Weight (kg), mean ± SD	8.0 ± 0.93	7.8 ± 0.92	0.69^b^	0.21
Feeding time before vaccination (minutes), mean ± SD	58.5 ± 32.7	58.7 ± 47.2	0.51^c^	0.01
Sleeping time before vaccination (minutes), mean ± SD	90.1 ± 39.0	87.1 ± 61.7	0.43^c^	0.05
Previous experience with painful procedures, *N* (%)			0.36^c^	0.10
Yes	26 (54.2)	22 (55)		
No	14 (43.8)	18 (45)		

^a^Chi-square test. ^b^Independent *t*-test. ^c^Chi-square test.

**Table 2:** Comparison of mean pain intensity and duration of crying in study groups.

Group	Intervention group (*n* = 40)	Control group (*n* = 40)	Test results	Effect size
	Mean ± SD	Mean ± SD		
Intensity of pain	6.1 ± 1.8	7.0 ± 2.1	*p*=0.032; *z* = −2.1^a^	0.46
Crying duration (seconds)	32.47 ± 16.7	51.02 ± 25.9	*p*=0.0001; *t* = 3.8^b^	0.85

^a^Mann–Whitney test. ^b^Independent *t*-test.

## Data Availability

The datasets used and analyzed during the current study are available from the corresponding author upon reasonable request.

## Supplementary Materials

Supplementary Materials CONSORT flow diagram 1: the CONSORT flow diagram of the study.
